# Using Paid Radio Advertisements to Promote Physical Activity Among Arkansas Tweens

**Published:** 2005-10-15

**Authors:** David Rath, Appathurai Balamurugan, Ernest J Oakleaf

**Affiliations:** HealthyArkansas Initiative, Arkansas Department of Health and Human Services; Senior Epidemiologist, Arkansas Department of Health and Human Services, Assistant Professor, University of Arkansas for Medical Sciences College of Public Health, Little Rock, Ark; Opinion Research Associates, Inc, Little Rock, Ark

## Abstract

**Introduction:**

The level of physical activity among children is a growing concern. Evidence shows that many children aged 9 to 13 years (tweens) do not participate in any organized physical activity during their nonschool hours, and some do not engage in any free-time physical activity. Physical inactivity is associated with a host of chronic diseases such as diabetes and cardiovascular disease. Paid media advertisements have been an effective method of promoting physical activity.

**Methods:**

From March 10, 2003, through June 29, 2003, we aired paid radio advertisements in six major Arkansas metropolitan areas to promote physical activity among tweens. In September 2003, we surveyed 295 Arkansas tweens by telephone to assess their exposure to the advertisements and the impact of the advertisements on their intent to participate in physical activity. In the same telephone survey, we also asked questions about the respondents' physical activity level. The data were weighted so that the results would be representative of the areas surveyed. The statistical analysis was performed using SPSS, version 11.5 (SPSS Inc, Chicago, Ill).

**Results:**

Of the tweens surveyed, 56.4% (95% confidence interval [CI], 50.7%–62.1%) reported hearing the radio advertisements. Of the tweens who heard the advertisement messages, 76.1% (95% CI, 69.4%–82.8%) said the messages made them more likely to get involved in physical activity. Younger tweens (aged 9 and 10 years) were less likely to have heard the advertisements than older tweens (aged 11 to 13 years). However, the advertisements were more likely to cause younger tweens to want to get involved in physical activity (odds ratio [OR] = 6.89, *P* = .003) than older tweens. Of the tweens surveyed, 74.9% (95% CI, 70.0%–79.8%) reported that they were involved in nonschool-sponsored sports, and 45.3% (95% CI, 39.6%–51.0%) were involved in school-sponsored sports.

**Conclusion:**

Paid media advertisements may be an effective way to promote physical activity among tweens. More rigorous evaluations of the impact of paid media advertisements among tweens, with larger samples and additional media markets, are needed. Future physical activity promotion efforts should focus on encouraging participation in school-sponsored sports and creating community environments conducive to physical activity.

## Introduction

Physical activity among children is a growing concern. According to the Centers for Disease Control and Prevention's (CDC's) Youth Media Campaign Longitudinal Survey, 61.5% of children aged 9 to 13 years, or *tweens,* do not participate in any organized physical activity during their nonschool hours, and 22.6% do not engage in any free-time physical activity (1). A similar trend is found among children aged 14 to 17 years (2,3). According to one study, increased television viewing is one of the primary causes of physical inactivity among children (4). Community issues such as a lack of infrastructure and parental safety concerns (5) also contribute to physical inactivity among children. Physical inactivity can lead to overweight and obesity, and the prevalence of overweight among children aged 2 to 19 years has been increasing (6). In addition to being linked to overweight and obesity, physical inactivity is a risk factor for a host of chronic diseases (7). The combination of physical inactivity and an unhealthy diet is the second leading cause of preventable death in the United States (8).

Two strategies that have been used to promote physical activity among children are physical education classes at school and mass media campaigns. School-based physical education classes can play a significant role in increasing participation in physical activity and help students gain the knowledge, attitudes, and skills required to be physically active (9,10). The prevalence of enrollment in physical education classes and the level of physical activity during physical education class has not increased among high school students since 1991 (2). (Similar data for tweens are not available.) ^ ^Mass media campaigns have been used successfully to promote physical activity (11,12). A systematic review of various interventions to increase physical activity revealed that mass media campaigns play a significant role in creating an awareness of the need for physical activity among children (13).

We assessed the impact of paid radio advertisements on promoting physical activity among Arkansas tweens. We used a telephone survey to evaluate the impact of our campaign on Arkansas tweens and assess the physical activity status of the participants.

## Methods

In 2003, the Arkansas Department of Health requested and received funds from the Tobacco Master Settlement Agreement for state health initiative proposals that met best-practice guidelines. The agreement, which was signed in 1998 by 46 state attorneys general, concluded a 4-year legal battle between states and the tobacco industry. The Arkansas Department of Health used some of these funds to develop programs to promote physical activity among tweens.

We obtained permission to locally air recordings of select radio advertisements from the CDC's VERB campaign (available from www.cdc.gov/youthcampaign/index.htm), a nationwide social marketing campaign that promotes physical activity among tweens through paid media advertisements, community programs and partnerships, and interactive media (14,15). We hired a media consultant to determine which radio channel markets were appropriate for our target audience. Based on the consultant's assessment, we chose radio channel markets from six major Arkansas metropolitan statistical areas, which combined represent 46.8% of the state's total population: Fayetteville/Rogers/Bentonville, Jonesboro, Texarkana, Little Rock/North Little Rock, Fort Smith, and El Dorado. It is estimated by the U.S. Census Bureau that 25.7% of the population in these metropolitan areas is younger than 18 years.

The radio channels in the designated markets were selected according to station rankings obtained from Arbitron's fall 2002 radio report for each market (available from www.arbitron.com/radio_stations/home.htm). The radio advertisements were aired on channels with music frequently listened to by tweens, such as contemporary, urban contemporary, and young country music. The advertisements were aired on the chosen stations in all six media markets from March 10, 2003, to June 29, 2003. To effectively reach the target population, the 60-second radio advertisements were aired every hour after 3:00 pm, when the tweens were usually home from school, and were not aired after 12:00 am. Because the schools were closed for summer vacation during May and June, the advertisements were aired hourly between 6:00 am and 11:59 pm during these months.

We assessed whether the target population heard the radio advertisements and whether those who heard the message changed their attitudes about becoming involved in physical activity. In addition, we assessed the physical activity level of the participants so that we could establish baseline information for our target population, which will assist us in planning future programs and policies for tweens.

Although several assessment tools to evaluate physical activity in children have been researched, self-report is the principal assessment tool used in many studies involving children (16). The Arkansas Department of Health developed a survey to meet our study objectives. Opinion Research Associates, Inc conducted the telephone survey (a cross-sectional study) and called 295 tweens in September 2003. The telephone numbers were obtained from a comprehensive database compiled from Arkansas's white-page telephone directories. Information on households with members in the target age range (9 to 13 years) was obtained from various data sources, such as school registration lists, magazine subscription lists, driver's license information, and voter registration lists. A random sample was drawn from the six media markets in Arkansas so that the results would be representative of the media markets in which the radio advertisements were aired. The survey questionnaire was pilot tested among tweens in the media markets, and appropriate changes were made based on the responses. Parental consent and assent from the tweens were obtained for the survey.

The survey questions were designed to determine whether the target audience had heard the campaign message and to measure its impact on the tweens (Appendix). For example, one question was, "Have you heard any VERB radio advertisements?" If the answer was yes, the person was asked, "What do you recall about the ad?" and "Do you feel the ad made you more likely to get involved in a physical activity?" The survey administrator recorded verbatim responses to the question about recalling the ad. The survey also included questions about physical activity status.

The data were weighted so that the results would be representative of the metropolitan statistical areas surveyed. The statistical analysis was performed using SPSS, version 11.5 (SPSS Inc, Chicago, Ill). We calculated 95% confidence intervals (CIs) for all frequencies and reported odds ratios (ORs) for all comparisons.

## Results

We surveyed 295 tweens by telephone (Table 1). Of the survey respondents, 89.2% were white, 10.8% were in other racial groups, 40.7% were female, and 59.3% were male.

Of the 295 tweens surveyed, 56.4% said that they had heard the advertisements on the radio (95% CI, 50.7%–62.1%) (Table 2). Of these, 68.7% (95% CI, 61.6%–75.8%) recalled the advertisements. When asked what they recalled, some of the frequently reported responses were "be active," "get out and play," "lots of verbs," "swim," "skate," and "it's what you do" (the tag line for the CDC VERB campaign). Of the tweens who heard the advertisements, 76.1% (95% CI, 69.4%–82.8%) said that the advertisements made them more likely to get involved in physical activity.

Respondents were also asked about their physical activity level, and 79.3% (95% CI, 74.8%–83.8%) said that they had taken physical education classes in the previous school year (Table 3). Only 45.3% (95% CI, 39.6%–51.0%) said that they had participated in school-sponsored sports or other physical activities during the school year. The most commonly reported school-sponsored sports played were basketball, football, soccer, track, and baseball. Of the surveyed tweens, 74.9% (95% CI, 70.0%–79.8%) said they participated in organized sports that were not school sponsored; baseball, basketball, swimming, soccer, and bike riding were commonly reported sports.

Compared with older tweens (11 to 13 years), younger tweens (9 and 10 years) were less likely to have heard the radio advertisements (OR 0.50, *P* = .007) (Table 4). However, younger tweens who heard the advertisements were more likely than older tweens to be motivated to get involved in a physical activity because of the advertisements (OR 6.89, *P* = .003). No significant difference in recalling the advertisements was found between younger and older tweens. In addition, no significant difference between younger and older tweens was found in physical education class enrollment or participation in organized school-sponsored sports or nonschool-sponsored sports. No sex or race difference was found in physical activity status, exposure to or recall of the advertisements, or level of motivation from the advertisements.

## Discussion

Because 76% of the tweens who heard the radio advertisements said that the advertisements motivated them to participate in physical activity, the results suggest that paid radio advertisements may be an effective way to promote physical activity among tweens. In addition, 69% of the tweens exposed to the advertisements recalled them during the survey. Studies have shown that positive health messages, such as the VERB advertisements, are generally recalled and likely to change behavior (17). When their subjective norms and beliefs are changed, tweens go through a phase of *intending* to do physical activity before actually *engaging* in physical activity (18); intentions correlate with actions (19). The ultimate success of our study will depend on whether it has long-term effects — involving tweens in physical activity and then helping them sustain it. Parental support and access to physical activities will help translate intentions into actions.

Younger tweens heard the advertisements less frequently than older tweens, so new strategies are needed to disseminate the radio advertisements to younger tweens. In addition, the advertisements did not motivate older tweens as much as younger tweens to become active, so new ways to motivate the older tweens are also needed. Future research should focus on identifying the aspects of radio advertisements that have universal appeal for younger and older tweens.

School-based physical education classes are strongly recommended as an approach to increasing physical activity among children and adolescents (9). Establishing policies and modifying curricula to increase the time spent in physical education classes will improve the level of physical activity among tweens. Participation in all types of physical activity decreases dramatically as age and grade in school increases (2). Evidence indicates that school-based interventions to increase physical activity through physical education classes are highly effective (20). By increasing students' physical activity level, policies mandating school-based physical education classes among tweens may consequently decrease the prevalence of overweight.

Three *Healthy People 2010* (21) objectives focus on increasing the daily required physical education for all students and spending at least 50% of school physical education class time actually being physically active. In addition, promoting school-sponsored sports that are already popular among children will increase participation in physical activities. The school sports most commonly played by tweens in our study (i.e., basketball, football, soccer, track, and baseball) are similar to those reported by tweens in previous studies (22). School-sponsored sports and other physical activities have been shown to be effective strategies for primary prevention of certain chronic diseases (23). Other studies have also shown that improved physical education may increase physical activity levels and provide health benefits to students (24,25), and interventions that involve changing school environments and policies have been shown to increase physical activity (26). There was no significant difference between younger and older tweens in school-sponsored sports participation during the school year or summer. Children and adolescents are at risk for becoming sedentary as they grow older (27). Because they spend most of their time in school, the type and amount of physical activity encouraged in school provides the foundation for a healthy lifestyle.

Seventy-five percent of tweens said that they participate in nonschool-sponsored organized sports during the school year or summer. The CDC's guidelines for school and community programs suggest that developmentally appropriate community sports and recreation programs that are appealing to youth, as well as physical and social environments that encourage and enable physical activity, are crucial to promoting physical activity outside of the school setting (28). In addition, social support from family and friends may promote participation in physical activity. Because much physical activity occurs outside the school setting, safe communities are essential (29). Creating safer communities and neighborhoods for children and adolescents is essential for encouraging and sustaining their physical activity levels.

The study has some limitations. The findings are representative of tweens with telephones who live in the six Arkansas metropolitan statistical areas. The self-report design could have affected our results because socially desirable responses tend to be overreported and therefore may be less accurate. The survey was conducted in September, so seasonal variations in sports participation might have influenced the results. In addition, although our radio campaign was not a part of CDC's VERB national media campaign, it was impossible to differentiate the impact of our efforts from those of the CDC campaign.

Although the results indicate that paid radio advertisements encourage Arkansas tweens to become involved in physical activity, the small sample size and testing of few media markets limit the generalizability of the results. More rigorous evaluations of the impact of paid media advertisements among tweens, with larger samples and more media markets, are needed. Developing innovative strategies to encourage and engage tweens in school-sponsored sports and creating conducive community environments to promote nonschool-sponsored sports are essential to promote physical activity among tweens.

## Figures and Tables

**Table 1:** Characteristics of Surveyed Arkansas Tweens (N = 295), 2003

**Characteristics**	**No. Respondents**	**% Respondents**

**Age**		

9-10 years (younger tweens)	95	32.2
11-13 years (older tweens)	200	67.8

**Sex**		

Female	120	40.7
Male	175	59.3

**Race**		

White	263	89.2
Black	22	7.5
Other	10	3.3

**Table 2:** Exposure to and Recall of Paid Radio Advertisements Among Surveyed Arkansas Tweens (N = 295), 2003

**Survey Responses**	**% Respondents (95% CI)^a^**
Heard radio advertisements	56.4 (50.7-62.1)
Recalled radio advertisements	68.7 (61.6-75.8)
Said that advertisements made them more likely to get involved in physical activity	76.1 (69.4-82.8)

a CI indicates confidence interval.

**Table 3:** Physical Activity Status Among Surveyed Arkansas Tweens (N = 295), 2003

**Survey Responses**	**% Respondents (95% CI)^a^**
Took physical education classes	79.3 (74.8-83.8)
Participated in school-sponsored sports	45.3 (39.6-51.0)
Participated in nonschool-sponsored sports	74.9 (70.0-79.8)

a CI indicates confidence interval.

**Table 4:** Survey Responses of Younger Tweens Compared With Older Tweens, Arkansas, 2003

**Survey Responses**	**Younger vs Older Tweens** ** OR (95% CI)^a^**	***P* Value**

**Paid radio advertisements**		

Heard advertisements	0.50 (0.30-0.83)	.007
Recalled advertisements	0.84 (0.39-1.80)	.65
Said that advertisements made them more likely to get involved in physical activity	6.89 (1.57-30.17)	.003

**Physical activity status**		

Took physical education classes	1.40 (0.72-2.70)	.32
Participated in school-sponsored sports	0.70 (0.42-1.16)	.16
Participated in nonschool-sponsored sports	1.25 (0.69-2.25)	.47

a OR indicates odds ratio; CI, confidence interval. Older tweens are the reference.

## Appendix. Survey Questions

**1. Have you heard any VERB radio announcements or advertisements encouraging physical activity?**

    1. Yes (*Go to questions 2 and 3.*)
    2. No (*Skip to question 4.*)
    (*DNR*) 9. Don't know/not sure/no response
    [*Editor's note: *DNR indicates did not respond.]

**2. What do you recall about the ad?** (*Record response.*) 

*Code:*

1. Something specific mentioned

2. Can't recall anything specific

**3. Do you feel the ad made you more likely to get involved in a physical activity?**

     1. Yes
    2. No
    (*DNR*) 9. Don't know/not sure/no response

**4. In the school year that ended last spring, did you take any physical education classes, that is, PE classes?**

    1. Yes
    2. No
    (*DNR*) 9. Don't know/not sure/no response

**5. Did you participate in any organized sports or other physical activities during the school year or over the summer that were sponsored by your school?**

    1. Yes (*Go to question 6.*)
     2. No (*Skip to question 7.*)
    (*DNR*) 9. Don't know/not sure/no response

**6. Which sports or activities were those?**(*Do not read. Code all that apply. Prompt if necessary.*)

*Code:*

1. Participated

2. Did not participate

- 9A1. Football
- 9A2. Basketball
- 9A3. Swimming
- 9A4. Track
- 9A5. Cheerleading
- 9A6. Drill team
- 9A7. Golf
- 9A8. Baseball, softball
- 9A9. Soccer
- 9A10. Tennis
- 9A11. Gymnastics
- 9A12. Skateboarding
- 9A13. Dance
- 9A14. Bike riding
- 9A15. Volleyball
- 12A16. Rollerblading, skating
- 12A17. Ice skating
- 12A18. Workout at gym, athletic club, or YMCA
- 12A19. Tae kwon do, karate, other martial arts
- 12A20. Other (*Record response.*)

**7. Did you participate in any organized sports or other physical activities during the school year or over the summer that were *not* sponsored by your school, that is, outside of school?**

    1. Yes (*Go to question 8.*)
    2. No (*Skip to question D1.*)
    (*DNR*) 9. Don't know/not sure/no response

**8. Which sports or activities were those?**

(*Do not read. Code all that apply. Prompt if necessary.*)

*Code:*

1. Participated

2. Did not participate

- 9A1. Football
- 9A2. Basketball
- 9A3. Swimming
- 9A4. Track
- 9A5. Cheerleading
- 9A6. Drill team
- 9A7. Golf
- 9A8. Baseball, softball
- 9A9. Soccer
- 9A10. Tennis
- 9A11. Gymnastics
- 9A12. Skateboarding
- 9A13. Dance
- 9A14. Bike riding
- 9A15. Volleyball
- 12A16. Rollerblading, skating
- 12A17. Ice skating
- 12A18. Workout at gym, athletic club, or YMCA
- 12A19. Tae kwon do, karate, other martial arts
- 12A20. Other (*Record response.*)

**D1. What is your age?** (*Record actual age reported.*)

**D2. To make sure we have a representative sample, could you tell me your ethnicity?Would you describe yourself as . . .**

    1. White or Caucasian
    2. Black or African-American
    3. Hispanic or Latino
    4. Asian or Pacific Islander
    5. American Indian or Native American Indian
     6. Other
*    (DNR) *9. Don't know/refused

** Thank you so much for your time, and good night!**

*Observe and classify:*

**D3. County** (*Code from number sheet.*) | ***0*** | ***5*** |__|__|__|

**D4. Sex**

1. Male
2. Female
